# Poultry Consumption and Human Cardiometabolic Health-Related Outcomes: A Narrative Review

**DOI:** 10.3390/nu15163550

**Published:** 2023-08-11

**Authors:** Gavin Connolly, Wayne W. Campbell

**Affiliations:** Department of Nutrition Science, Purdue University, West Lafayette, IN 47907, USA; connolg@purdue.edu

**Keywords:** chicken, turkey, protein, animal-based, animal protein, cardiovascular, type 2 diabetes mellitus, metabolic disease

## Abstract

Poultry meats, in particular chicken, have high rates of consumption globally. Poultry is the most consumed type of meat in the United States (US), with chicken being the most common type of poultry consumed. The amounts of chicken and total poultry consumed in the US have more than tripled over the last six decades. This narrative review describes nutritional profiles of commonly consumed chicken/poultry products, consumption trends, and dietary recommendations in the US. Overviews of the scientific literature pertaining to associations between, and effects of consuming chicken/poultry on, body weight and body composition, cardiovascular disease (CVD), and type II diabetes mellitus (T2DM) are provided. Limited evidence from randomized controlled trials indicates the consumption of lean unprocessed chicken as a primary dietary protein source has either beneficial or neutral effects on body weight and body composition and risk factors for CVD and T2DM. Apparently, zero randomized controlled feeding trials have specifically assessed the effects of consuming processed chicken/poultry on these health outcomes. Evidence from observational studies is less consistent, likely due to confounding factors such as a lack of a description of and distinctions among types of chicken/poultry products, amounts consumed, and cooking and preservation methods. New experimental and observational research on the impacts of consuming chicken/poultry, especially processed versions, on cardiometabolic health is sorely needed.

## 1. Introduction

Poultry meats, with chicken being the predominant type of poultry consumed, is the most consumed type of meat in the United States (US) [1]. Central to providing high-quality protein and other nutrients, poultry meats are generally considered as healthy [2]. Poultry meats, in particular chicken, are relatively affordable and easily accessible resulting in high rates of consumption globally [3] and in the US [1].

In 2022, we published a systematically searched scoping review pertinent to poultry intake and all facets of human health [4]. Among 13,141 articles identified, 525 met inclusion criteria. Among these 525 articles, 41 were on cardiovascular disease (CVD) morbidity and mortality; 52 on CVD risk factors; 32 on type II diabetes mellitus (T2DM) morbidity and mortality; 33 on T2DM risk factors; and 42 on body weight and body composition [4]. This scoping review did not present results described in the articles. The findings and articles included from the scoping review, along with more recently published articles, were used as a foundation for this narrative review on poultry consumption and human cardiometabolic health-related outcomes. Overviews of the scientific literature pertaining to associations between, and effects of consuming varying amounts and types of poultry on body weight and body composition, CVD, and T2DM are provided.

## 2. Nutritional Content of Chicken/Poultry

Chicken and other poultry meats provide macronutrients and micronutrients considered essential for human health and physiological functioning [5,6]. Essential nutrients are compounds the human body cannot make or cannot make in sufficient quantities and must be obtained via dietary sources. Chicken and other poultry-based proteins, like all other animal-based proteins such as other meats, milk, and eggs, as well as plant-based soy protein, are considered high-quality complete protein sources [5]. These foods provide a full complement of all 20 amino acids and adequate quantities of the nine essential amino acids.

Chicken and other poultry products have varying energetic and nutrient profiles and do not naturally contain carbohydrates (Table 1). Chicken contains 23–31 g of protein per 100 g or 3.5 oz, depending on the cut of chicken. The total fat content can vary from 3.6–2.1 g per 100 g, or 3.5 oz, in the leanest cuts such as cooked skinless chicken breast and cooked skinless turkey breast, respectively, to 16.9 g per 100 g/3.5 oz in cooked chicken wings with the skin. For example, cooked skinless chicken and turkey breast have 19% and 13%, respectively, of total energy content from fat, while cooked chicken wings with skin have 60%. The proportion of total fat from saturated fat is consistently about 27–29% (1–5 g per 100 g serving) for various cuts of chicken. Monounsaturated and polyunsaturated fats are about 32–46% and 20–23% (1.2–7.7 g and 0.8–3.6 g per 100 g serving), respectively, of the total fat content for different cuts of chicken [7].

Chicken and other poultry products also provide essential nutrients that are commonly under-consumed, including the minerals magnesium, phosphorous, potassium, selenium, and iron, and the B-group vitamins including thiamin (B1), riboflavin (B2), niacin (B3), pantothenic acid (B5), pyridoxal (B6), cobalamin (B12), and choline [6]. Unprocessed chicken and other poultry products are naturally low in salt. Chicken and other poultry products can be purchased as fresh unprocessed cuts (no preservation techniques other than refrigeration or freezing) or as processed items preserved by smoking, curing, salting, and/or the addition of chemical preservatives [6,8]. As sodium is a common preservative added to chicken and other poultry products to prolong shelf life and/or as a flavor enhancer, processed chicken and other poultry products may contain high or very high amounts of sodium [5]. As such, the sodium content of unprocessed and processed chicken and other poultry products can vary from <100 mg per 100 g serving in fresh, cooked, cuts of chicken to >2000 mg per 100 g serving in turkey bacon. The process of curing meats utilizes both NaCl and a synthetic nitrate or nitrite salt to preserve meat by inhibiting bacterial growth [9]. Many processed poultry deli meats contain either synthetic nitrate or nitrite salts, or nitrates from natural sources such as celery root powder. The addition of these preservatives can catalyze the formation of carcinogens, notably nitrosyl heme, when added to meat products that contain heme iron [9].

## 3. Chicken and Poultry Consumption in the US

**Figure 1:**
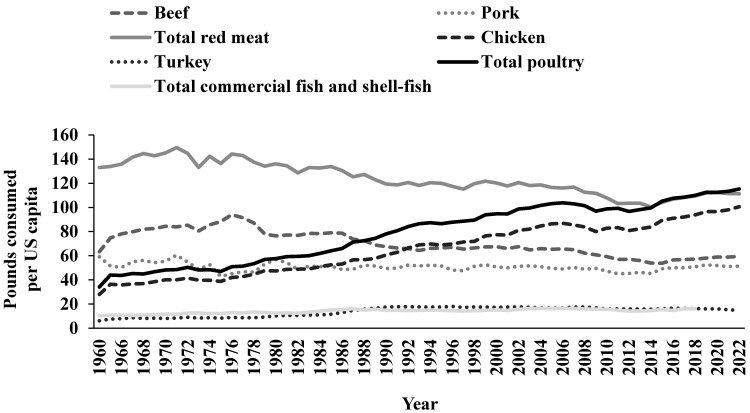
Pounds consumed per US capita for meats, fish, and shellfish from 1960 to 2022. Source: This figure was created using United States Department of Agriculture open-source data presented in tabular form by the National Chicken Council [1].

Chicken meat is the most consumed meat in the US per capita [1]. The US has the largest broiler chicken industry in the world and more chicken is consumed in the US than in any other country. In 2022, annual consumption of chicken and turkey in the US per capita was estimated to be 100.6 pounds (lb) and 14.7 lb, respectively, for a total of 115.3 lb of poultry. In comparison, annual consumption of beef and pork was 59.4 lb and 51.4 lb per capita, respectively, for a total of 111.3 lb of red meat. Total consumption of commercial fish and shellfish was 16.1 lb per capita (last reported in 2018) [1]. From 1960 to 2022, chicken and total poultry consumption steadily increased and more than tripled. In contrast, pork consumption remained stable and beef and total red meat intakes have decreased (Figure 1).

In the US, the National Health and Nutrition Examination Survey (NHANES) is a nationally representative sample of 5000 individuals per data collection period which assesses the health and nutritional status of adults and children. Based on NHANES data, protein intake is estimated to be 97 g/d and 67 g/d for males and females, respectively [10], which for both sexes equate to about 16% of total energy intake [11]. Animal-derived protein sources account for about two-thirds of total protein intake [10,12]. Based on an overall average intake of 83 g protein/d, poultry intake is estimated to be 12.5 g/d or 0.44 oz/d.

Although poultry was the top source of total dietary protein intake (10%), it ranked 6th for total dietary energy intake (<4%) [13]. More specifically, data from 2007 to 2010 indicate chicken whole pieces were the number one source of animal protein, accounting for 13.9% of total animal protein intake, 7.2% of total protein intake, and 2.8% of total energy intake. Turkey, duck, and other poultry ranked 17th, accounting for 1.9% of total animal protein intake, 0.9% of total protein intake, and 0.3% of total energy intake [10].

## 4. Dietary Guidance in the United States

The estimated average requirement (EAR) for total protein is 0.66 g/kg/d for men and women 19+ years of age The EAR is assumed to meet the protein requirement of 50% of the healthy adult population. The recommended dietary allowance (RDA) for total protein is 0.8 g/kg/d, which is two standard deviations above the EAR. The RDA is assumed to meet the protein needs of 97.5% of healthy adults 19+ years of age.

The Dietary Guidelines for Americans (DGA), updated and re-published every 5 years since 1980, serves as the evidence-based foundation for nutrition education by the US Federal Government to inform the development of food, nutrition, and health policies and programs. The intended audiences for the DGA are policymakers and health professionals. A primary focus of the DGA is disease prevention [6]. The 2020–2025 DGA states that “common characteristics of dietary patterns associated with positive health outcomes include relatively higher intakes of vegetables, fruits, legumes, whole grains, low- or non-fat dairy, lean meats and poultry, seafood, nuts, and unsaturated vegetable oils, and relatively lower consumption of red and processed meats, sugar-sweetened foods and beverages, and refined grains” [6].

The 2020–2025 DGA provides examples of three healthy dietary patterns. The three dietary patterns are the Healthy US-Style Dietary Pattern, the Healthy Mediterranean-Style Dietary Pattern, and the Healthy Vegetarian Dietary Pattern. In 2011, the DGA released the MyPlate icon to support the recommendations and translation of healthy dietary patterns. The MyPlate icon is a visual representation of a plate sectioned into the major food groups to aid individuals in choosing recommended amounts and types of healthy foods at mealtimes. The major food groups represented on the plate are vegetables, fruits, grains, and protein, accompanied by another circle for dairy.

Regarding the Protein Foods Group, the DGA provides guidance on protein foods from animal and plant sources in nutrient-dense forms, based on quantities measured in ounce-equivalents as defined by the United States Department of Agriculture [6,14]. The recommendation for protein foods in the Healthy US-Style Dietary Pattern at the 2000 kcal/d energy level is 5.5 ounce-equivalents of protein foods per day. The Protein Foods Group consists of meat (red), poultry, seafood, eggs, nuts and seeds (including nut and seed butters), legumes (beans and peas), and soy products (excluding calcium added soy milk assigned to the Dairy Group, and raw, green soybean assigned to the Vegetables Food Group) [6,14].

The Meat and Poultry components are further subdivided into Meat, Poultry, Organ Meat, and Cured Meat. The Meat component includes red meats such as beef, goat, lamb, pork (includes fresh or uncured ham), veal, and game meat (e.g., bear, bison, moose, opossum, rabbit, raccoon, squirrel, venison). The Poultry component includes chicken, Cornish hens, dove, duck, game birds (e.g., ostrich, pheasant, quail), goose, and turkey. The Cured Meat component includes cured or smoked meat products such as frankfurters, sausages, and luncheon meats, and cured meat made from beef, chicken, pork, and turkey. The Organ Meat component includes brain, chitterlings, giblets, gizzard, heart, kidney, stomach, sweetbreads, thymus, tongue, and tripe [14].

Supporting the 2020–2025 key health recommendations, individuals who consume omnivorous dietary patterns are encouraged to choose lean meats and animal products (<10 g of total fats and ≤4.5 g of saturated fats). Since processed meats and poultry (e.g., luncheon meats, bacon, sausages, beef jerky) are generally high sodium foods, limiting their intake will help meet the 2300 mg/d sodium intake recommendation [6]. Poultry (excluding deli and mixed dishes) contributes an estimated 4% of total saturated fat and 5% of sodium intakes in the US [6]. It is postulated that limiting red meat and poultry products high in saturated fat and sodium will be beneficial in limiting total energy, saturated fat, and sodium intakes. Currently, most of the US population consume these dietary components in excess [6]. The incorporation of lean, unprocessed chicken and other poultry products will provide individuals with high-quality sources of dietary protein and important nutrients, including heme-iron, selenium, and niacin, when consumed within energy requirements [6].

## 5. Experimental Approaches and Considerations When Studying the Influence of Poultry Intake on Human Health

### 5.1. Experimental Approaches

Both observational and experimental human trials provide valuable information regarding dietary choices and human health. However, findings from these studies do not always result in the same conclusions. Observational studies provide associations between a variable of interest and an outcome. These types of studies generally have large sample sizes, as they may be conducted at a population level, in highly diverse populations, and include long follow-up periods. While these observations can be insightful, potential confounding and uncontrolled variables preclude establishing a cause-and-effect relationship. Randomized controlled trials (RCTs) are generally considered the “gold standard” to assess a variable of interest and a specific outcome. In RCTs, participants are randomized to either an experimental intervention or a non-experimental or alternative intervention control group, and the outcome of interest is compared between groups. RCTs aim to keep as many variables—as is feasible—the same between groups, thereby reducing the risk of bias and the effects of confounding. Importantly, RCTs have relatively smaller sample sizes compared to observational studies. They also may lack generalizability and real-world application. Observational studies may assess endpoints, such as disease onset or death, whereas RCTs generally assess intermediary risk factors for disease [15]. For a more detailed description and comparison of these study designs, the reader may refer to articles addressing this topic by Hébert et al. [16], Booth and Tannock [17], Faraoni and Schaefer [18], and Barton [19].

Observational and experimental studies may be used to investigate the influences of individual nutrients, individual foods, or overall dietary patterns on human health [15]. Humans do not consume nutrients or foods in isolation, but within a dietary pattern dependent upon their available resources and/or personal preferences. This hierarchy of consumption—nutrients, foods, and patterns—will be applied in the following sections in addressing how chicken and/or other poultry consumption influence body weight and body composition, CVD, and T2DM. When limited evidence is available pertaining to chicken, we will supplement it with information pertaining to total poultry consumption. The definition of poultry included within this review is based on and consistent with the definition for the Poultry Food Group in the DGA 2020–2025; stating, “poultry includes chicken, Cornish hens, dove, duck, game birds (e.g., ostrich, pheasant, quail), goose, and turkey” [6].

### 5.2. Potential Explanatory Variables

Published research on poultry is complicated by the lack of a clear definition used among studies for poultry, a lack of reporting on whether the poultry products were unprocessed or processed, and cooking methods, all of which influence human health outcomes [20,21].This is likely attributable to using food frequency questionnaires to assess dietary intake in observational studies [22] along with broad food categories [20,23].

#### 5.2.1. Definitions

Poultry and health research is hampered by the lack of a clear definition used among studies. A 2020 systematic review and landscape analysis by O’Connor et al. [20], determined that of 52 identified studies assessing poultry consumption and health outcomes, only 63% provided a definition for poultry. In addition, poultry is often defined as “white meat”, which may or may not also include fish and/or rabbit, or the definition of poultry includes rabbit. While “rabbit” should be considered a red meat, it is commonly included as poultry due to similarities in nutrition profiles [24,25]. These broad and inconsistent classifications make it problematic when trying to determine the influence of such foods on health outcomes. Classifying poultry, fatty fish, and lean fish, as “white meat” does not account for differences in nutritional profiles [23]. For example, fatty fish, which is higher in omega-3 fatty acids, have cardioprotective benefits [26]. The commonly used classification of “white meat” that includes fatty fish, lean fish, and poultry, may lead to the cardioprotective benefits of fatty fish being attributed to lean fish and poultry products. Adding to confusion, poultry contains both “white” and “dark” meat, which differ in macronutrient composition.

#### 5.2.2. Processing

Processed meats, compared to unprocessed meats, contain high amounts of preservatives, such as sodium and nitrates. The sodium content of processed meats may be 400% higher than unprocessed meats, contributing to associations between processed meat intake and increased blood pressure [21,27]. Nitrates in processed meats may increase coagulation, inflammatory cytokines, and reactive oxygen species [28]. These indexes of endothelial cell dysfunction are involved in the process of atherosclerotic lesion manifestation and progression as well as increases in insulin resistance [29]. Heme-iron in meats increases oxidative stress and is associated with the atherosclerotic processes and cardiometabolic disease risk factors such as hypertension, hypercholesterolemia, endothelial dysfunction, insulin resistance, and T2DM [29,30,31]. Substituting white meats, especially unprocessed white meats, for red meats may reduce the risk of CVD [21] and all-cause mortality [32].

#### 5.2.3. Cooking Methods

Varied cooking methods of poultry products introduce variability in the concentrations of non-nutritive compounds. Heterocyclic aromatic amines are formed in cooked meats through a reaction between amino acids, creatine, and sugars. Burned or charred meats have higher levels of heterocyclic aromatic amines and benzopyrene in comparison to meats cooked at a lower temperature or are not visibly blackened. Barbequed and grilled poultry products contain higher concentrations of heterocyclic aromatic amines than baked or boiled products. Polycyclic aromatic hydrocarbons are formed when cooking meat products at high temperatures, particularly during the process of smoking, grilling, roasting, and frying [10,11]. High-heat cooking methods, such as frying and grilling, produce higher amounts of advanced glycation end-products. Prior to and following cooking, these highly oxidant glycotoxins are found in greater amounts in animal-based meats compared to carbohydrate-based foods such as fruits, vegetables, and whole grains, and dairy products [12].

Advanced glycation end products are linked with increased insulin resistance and blood glucose concentrations [29]. These compounds can increase the risk of adverse cardiometabolic health-related outcomes, as they decrease the vascular endothelium response by a reduction in vasodilation and increased oxidative stress and inflammation [33,34]. The consumption of chicken cooked by open flame and/or high temperature has been associated with a 15% increased risk of T2DM [35]. Frying and the frequency of fried chicken consumption, has also been associated with increased CVD mortality in older-aged women in the WHI [36] and an increased incidence of T2DM in the Black Women’s Health Study [37]. Along with advanced glycation end products, high-heat cooking methods, level of doneness, and processing of meats (which commonly use high-heat cooking methods) lead to the formation heterocyclic aromatic amines and polycyclic aromatic hydrocarbons, which are associated with an increased risk of CVD and T2DM [33].

## 6. Poultry Consumption and Body Weight and Body Composition

In the US, an estimated 73.6% of adults are classified as overweight (31.7%) or obese (41.9%) [38], while approximately 36% of children and adolescents aged 2–19 years are classified as overweight (16.6%) or obese (19.7%) [39]. Over a 20-year period (1999–2018) in the US, the prevalence of obesity and severe obesity have increased from 30.5% to 41.9%, and 4.7% to 9.2%, respectively [38]. Obesity may also lead to preventable and modifiable morbidities, such as CVD and T2DM, that are leading causes of premature death in the US and worldwide. Obesity comes with a large financial burden and obesity and obesity-related chronic diseases are a top priority for public health policy in the US. Obesity was estimated to cost the US at least $147 billion in direct medical costs in 2018 and another $4 billion in indirect costs, such as lost productivity. On an individual basis, people with obesity were estimated to have $1429 higher medical costs per year than people who were normal weight [40,41].

Diet and physical activity are two of the most effective ways to decrease the risk of or prevent overweight and obesity and related comorbidities [42,43,44]. Body weight and body composition are influenced by energy balance. Energy balance is determined by the relationship between energy intake via foods and beverages and energy expenditure via basal metabolic rate and the thermic effects of feeding and physical activity [42,45,46]. The relationship between energy intake and energy expenditure determines whether there is weight loss, weight maintenance, or weight gain [42,46]. Higher dietary protein intake, inclusive of chicken and/or other poultry products, may aid in weight control and favorable body composition changes, including decreased fat mass and/or increased lean body mass [47,48].

Evidence regarding weight management indicates the consumption of dietary protein and a higher protein diet can: (1) increase satiety; (2) increase thermogenesis; and (3) increase, maintain, or attenuate the loss of lean body mass [45,46,49,50]. The following sections document evidence from identified RCTs and observational studies specifically pertaining to chicken/poultry consumption on body mass and body composition.

### 6.1. RCTs

The available evidence from RCTs specific to chicken/poultry consumption and body weight or body composition, suggests chicken/poultry consumption has a neutral effect on body weight and body composition in the context of weight maintenance in adult men and women [51], and a favorable effect on body mass and body composition in the context of diet-induced weight loss in adult women classified as overweight [52] or with obesity [53]. Moreover, chicken protein in supplemental form in combination with resistance exercise training was beneficial for body composition changes in young adults [54].

A nine-month randomized cross-over trial by Murphy et al. [51] investigated the effects of regular consumption of pork, beef, or chicken on indices of adiposity via anthropometric and DEXA assessments. Forty-nine adults classified as overweight or obese were randomly assigned to consume five (for women) to seven (for men) 140–150 g per serving/week of pork, chicken, or lean beef by incorporating it into their habitual diets for the initial three-month period, followed by two more three-month periods consuming each of the alternative meats. There were no differences reported for energy or macronutrient intakes during each intervention period, indicating participants were substituting the meats in their diets without changing total energy or macronutrient intakes. The results showed no differences in body mass, BMI, any index of adiposity (% body fat, FM, abdominal fat, waist-to-hip ratio), or measures of lean mass among the pork, beef, or chicken diets. Consistent with the author’s hypothesis, purposefully consuming known amounts of different meats (lean pork, chicken, or beef) did not influence body weight or body composition after each three-month intervention period [51].

In a 12-week RCT, 61 women characterized as obese, middle aged, and moderately physically active consumed individualized 500 kcal/d energy deficit diets with 20% of total energy intake primarily from lean beef versus lean chicken [53]. The diets were also designed to be equivalent in terms of total energy and percentage of energy from fat with the primary outcomes being bodyweight and body composition (assessed by hydrodensitometry). The beef and chicken groups each reduced body weight (−5.6 ± 0.6 kg and −6.0 ± 0.5 kg, respectively (mean ± standard error (SE)) and body fat (−3.6 ± 3.3% and −4.1 ± 3.3%, respectively) from baseline to post-intervention, with no differences between groups [53].

Similarly, an RCT by Mahon et al. [52] investigated the effects of dietary protein intake on energy restriction-induced changes in body mass and body composition (assessed via DEXA) in 54 women classified as overweight and postmenopausal. For the nine-week intervention, three energy restricted groups consumed 1000 kcal/d of a lacto-ovo vegetarian base diet along with 250 kcal/d of either beef, chicken, or carbohydrate/fat foods; a non-interventional control group consumed their habitual diets. Energy intake was not different among the three energy restriction groups with total protein intake constituting 25% of total energy intake in the chicken and beef groups and 17% in the carbohydrate/fat group. Combined, the energy restricted groups decreased their body mass (−6.7 ± 2.4 kg; mean ± standard deviation (SD)), fat mass (−4.6 ± 1.9 kg, 13%), and fat-free mass (−2.1 ± 1.1 kg, 5%). There were no differences among the three energy restricted groups, except for body mass, as the chicken group lost more body mass than the carbohydrate/fat group: −7.9 ± 2.6 kg compared to −5.6 ± 1.8 kg, respectively [52].

The benefits of protein and higher protein intakes for body weight and body composition are well documented [45,46,49,50]. Dietary protein supplements are increasingly popular sources of protein [55]. Chicken and other forms of poultry can be produced as dietary protein supplements. Dairy- and plant-based proteins are typically used for research, often in combination with resistance exercise training [54]. Our literature search identified one double-blind, parallel, RCT with 41 young adult men (*n* = 19) and women (*n* = 22) investigating the effects (after eight weeks) of consuming beef protein isolate, hydrolyzed chicken, whey protein concentrate, and maltodextrin (as a control) consumption on resistance exercise training-induced changes in body composition [54]. Total energy intake and macronutrient intakes were not different among groups, with carbohydrate, fat, and protein intakes averaging 48%, 29%, and 23% of energy intake, respectively. This protein intake equates to 2.0–2.2 g/kg/d, with participants consuming a 46 g bolus of protein or control immediately post-exercise or at a similar time on non-training days [54].

There were no differences among groups for lean body mass and fat mass at baseline. The chicken, beef, and whey protein groups each increased lean body mass and decreased fat mass from baseline to post-intervention with no differences among groups. Interestingly, while total energy intake and total protein intake did not differ among the protein groups and control group, no changes in either lean body mass or fat mass were observed over time in the control group [54]. These findings indicate that consuming a higher protein diet, which may be obtained by the consumption of a supplemental form of chicken protein, aids in promoting increases in lean body mass and decreases in fat mass during resistance exercise training in young adults.

### 6.2. Observational Studies

Observational studies show that chicken/poultry consumption has either a positive or neutral association with body weight and BMI in adults or children and adolescents. An important consideration regarding observational studies is that inconsistencies in the findings may, in part, be explained by a lack of distinction and specific categorizations of chicken/poultry products (e.g., unprocessed vs. processed) and cooking methods, which influence the energy and nutrient profiles, and potentially, measures of body weight and body composition.

#### 6.2.1. Observational Studies in Adults

A prospective observational study of 3902 men and women aged 55–69 years from the Netherlands Cohort Study found no association between total meat intake and changes in BMI over a 14-year follow-up period. Subgroup analysis of different types of meat indicated men and women in the highest quintile for chicken intake (>22.8 g/d) had a greater increase in BMI (men: +0.19 kg/m^2^; women: +0.53 kg/m^2^) compared to those in the lowest quintile who consumed no chicken [56]. A cross-sectional analysis in the US of 508 men (mean age of 53.7 years) and 1293 women (mean age of 49.5 years) classified as obese showed greater consumption of fried chicken was associated with higher BMI, in both sex groups [57]. A cross-sectional analysis of a community-based cohort of 204 African American/Black men indicated 90% ate their chicken fried. Consuming fried chicken with skin was associated with higher BMI [58]. A cross-sectional analysis of 287 individuals classified as obese and 1871 individuals classified as normal weight, aged 18–68 years, in India, found individuals classified as obese consumed greater amounts of fried/grilled chicken compared to individuals classified as normal weight [59].

In contrast, there is also evidence of neutral associations between poultry consumption and body weight and body composition. A prospective observational study in a cohort of 1638 men and women aged 18–60 years examined associations between unhealthy eating behaviors and weight gain over a 3.5-year follow-up period [60]. The investigators reported no association with never/almost never removing skin from chicken and weight gain, compared to those who removed skin from chicken. Additionally, cross-sectional data from 418 adults and older adults showed no associations between poultry intake and BMI or waist circumference [61].

#### 6.2.2. Observational Studies in Children and Adolescents

Based on cross-sectional data from 1562 children aged 10 years from the Bogalusa Heart Study, no associations were observed between poultry consumption and overweight and obesity classifications [62]. Additionally, no associations were observed between poultry consumption and overweight and obesity classifications for ethnic−gender groups, namely, Euro-American males, Euro-American females, African-American males, and African-American females [62]. Harris et al. [63], using data from a prospective cohort study with adolescents, investigated associations between different types of meat with measures of body composition. They observed higher poultry intake in males at 10 years of age was positively associated with a higher fat mass index at 15 years of age. No associations were observed between poultry consumption and body composition changes in females [63].

The incongruence of these findings may be partially explained by lack of distinctions in the types and/or cooking methods of chicken/poultry consumed. More specific details on the type of poultry consumed were provided in the Avon Longitudinal Study of Parents and Children in the United Kingdom. Among the 4646 boys and girls aged 7–13 years of age, consuming greater amounts of coated (breaded or battered), but not uncoated poultry, was associated with excess weight gain [64]. The Harris et al. [63] study was done with all types of poultry products included, while the Dong et al. [64] study divided poultry products into coated and uncoated subcategories.

Cross-sectional analysis of data from 2525 freshmen university students in Turkey aged 18–22 years, revealed no association between chicken (excluding fried chicken) or burgers/fried chicken consumption and BMI [65]. In line with this finding, a cross-sectional analysis of data from 406 female students in Jordan, found consuming chicken more frequently (≥4 vs. <4 times/week) did not predict an increased likelihood of being classified as obese [66]. In contrast, cross-sectional data from 300 university students in Iran indicated consumption of fried chicken was associated with 40% increased odds of being classified as obese [67].

## 7. Poultry Consumption and CVD

Cardiovascular disease encompasses a group of heart and blood vessel disorders, including coronary heart disease (CHD), heart failure, cerebrovascular disease/stroke, and peripheral arterial disease [68,69]. CVD is the leading cause of diet-related deaths worldwide and deaths in the US, accounting for 9.5 million and greater than 877,500 deaths in 2017 [70] and 2020 [71,72], respectively. In the US, CVD accounts for approximately one third of all deaths and increases with age [71,73]. CVD comes with significant societal and economic burdens with direct medical costs for CVD estimated to be $216 billion, with an additional $147 billion in indirect costs, in 2020 [73,74]. As CVD is the leading cause of diet-related deaths worldwide, dietary components can differentially influence risk factors for CVD and the occurrence of CVD. The following sections will focus on evidence from RCTs and observational studies on the effects and associations of chicken/poultry consumption and CVD health-related outcomes.

### 7.1. RCTs

RCTs with lean chicken/poultry as the primary protein source in diets show neutral or beneficial effects on CVD risk factors.

A controlled-feeding RCT by Bergeron et al. [75] including 113 “generally healthy” adults (69 females, 34 males) investigated the effects of consuming a typical American diet with varying protein food sources and saturated fat intakes on blood lipids and lipoproteins. Protein sources were 12% of energy from lean poultry, lean red meat, or nonmeat sources and saturated fat intakes were 13% or 7% of energy intake. Participants were randomized to one of two parallel arms, either high- (*n* = 63) or low- (*n* = 52) saturated fat, and within each arm consumed either poultry, red meat, or nonmeat protein sources for four weeks in a three-period cross-over design, with a two-to-seven-week washout period between each intervention period. The higher-saturated fat intake was achieved primarily via the consumption of high-fat dairy products and butter.

Independent of saturated fat intake, consuming the nonmeat diet, but not the poultry or beef diet reduced total cholesterol, LDL cholesterol, non-HDL cholesterol, HDL cholesterol, apolipoprotein B, and apolipoprotein A1. Protein source did not influence triglycerides or total to HDL cholesterol ratio among the three diets. Independent of dietary protein source, higher-saturated fat intake resulted in increased total cholesterol, LDL cholesterol, and non-HDL cholesterol compared to lower-saturated fat intake [75]. These findings indicate that source of dietary protein and saturated fat intake each independently affect multiple CVD risk factors. The results also support the health promoting properties of consuming nonmeat protein foods compared to animal-based protein foods.

Two controlled-feeding RCTs have investigated the effects of lean chicken/poultry intake on blood lipids and lipoproteins in males with hypercholesterolemia [76,77]. In a randomized crossover design study by Beauchesne-Rondeau et al. [76] 17 white, weight-stable males classified as overweight and hypercholesterolemic (mean age ± SD of 50.1 ± 3.3 years) consumed an American Heart Association-style dietary pattern for 26-day periods. The pattern had a high polyunsaturated-to-saturated fatty acid ratio and a high fiber content as a base diet, as well as either lean poultry (skinless chicken and ground turkey), lean beef, or lean fish. Each experimental treatment period was separated by a six-week washout. The poultry, beef, and fish diets each reduced total cholesterol, LDL cholesterol, apolipoprotein B, total to HDL cholesterol ratio, and triglycerides from pre-to post-intervention, with no differences between interventions [76]. These findings suggest the health promoting properties of an American Heart Association-style diet may be achieved when the primary source of protein is lean poultry, beef, or fish.

Scott et al. [77] provide further support for these findings. In this RCT, 38 men with hypercholesterolemia consumed an American Heart Association-style diet with 85 g per 1000 kcal of lean chicken or lean beef for five weeks. Both diet groups reduced total and LDL cholesterol, with no differences between diets [77]. However, in contrast to Beauchesne-Rondeau et al. [76], Scott et al. [77] did not observe any changes in triglyceride concentrations or total to HDL cholesterol ratios for either diet. Taken together, the findings from these two RCTs indicate that when lean meats (chicken/poultry, red meat, or fish) are the primary protein food source consumed as part of this healthy dietary pattern, it can result in favorable changes in blood lipids and lipoproteins in males with hypercholesterolemia, most notably total and LDL cholesterol.

In the two RCTs previously described by Mahon et al. [52] and Melanson et al. [53], women classified as overweight and obese consumed energy restricted diets for 9–12 weeks, with chicken or beef as the primary protein source. Both diets decreased total and LDL cholesterol, with no differences between groups. Additionally, Mahon et al. [52] found no changes over time with the chicken or beef diet on triglycerides, HDL cholesterol, or C-reactive protein. Melanson et al. [53] also reported no differences in triglycerides from baseline to post-intervention for the chicken or beef groups and no differences between groups. Another randomized cross-over trial by Mateo-Gallego et al. [78] investigated the effects of consuming lean red meat (lean breed lamb) and lean chicken as part of an energy balanced diet, each for a period of five weeks, on lipid profiles in 36 older (mean age of 71 years) women classified as overweight and obese [78]. No differences were observed for total and LDL cholesterol, triglycerides, apolipoprotein B or lipoprotein (a) from baseline to post-intervention for the chicken or red meat diet, or between groups for any outcome [78].

### 7.2. Observational Studies

Evidence indicates chicken/poultry consumption has either neutral or beneficial associations with CVD morbidity and mortality, and neutral or adverse associations with blood pressure and hypertension.

#### 7.2.1. CVD

A 2021 systematic review and meta-analysis of 10 prospective cohort studies by Papp et al. [2] found no association between the highest and lowest intakes of poultry and risk for CVD or CHD. Linear dose response meta-analyses found no association per 100 g/d increase in poultry intake for CVD or CHD based on nine and 10 studies, respectively. Non-linear meta-analysis showed evidence for an association between poultry consumption and CVD, but not CHD [2]. These findings should be interpreted with caution, due to the certainty of evidence being deemed weak for CVD and CHD using the GRADE approach. A meta-analysis of prospective cohort studies by Abete et al. [30] found no relationship between poultry intake and CVD or ischemic heart disease mortality between the highest and lowest intakes or in the dose−response meta-analysis [30]. A pooled analysis of eight prospective cohort studies conducted in Asia found no associations between poultry consumption and CVD [79].

In line with these findings, several prospective cohort studies have shown no associations between poultry consumption and CVD. Analyses of two large Chinese population-based prospective cohort studies, The Shanghai Women’s Health Study (SWHS) and The Shanghai Men’s Health Study (SMHS), as well as The Japan Collaborative Cohort (JACC), showed no associations between poultry consumption and CVD mortality [80,81]. The Pan-European EPIC cohort, The JACC, and the Atherosclerosis Risk in Communities (ARIC) Study, showed that poultry consumption was not associated with risk of ischemic heart disease [81,82,83]. Similarly, the ARIC Study showed no associations between poultry consumption and peripheral arterial disease [84].

There is also evidence from prospective cohort studies that higher intakes of chick-en/poultry are associated with a decreased risk of CVD among men and women in a dose−response relationship [85] and CHD among women in the Nurses’ Health Study (NHS) with a 26-year follow-up [86]. The Korean Genome and Epidemiology Study (KoGES) showed a dose−response association between intakes of unprocessed chicken and decreased CVD risk. Those in the highest quintile of chicken intake were 32% less likely to develop CVD compared to those in the lowest quintile (1.41 vs. 0 median servings/week) [85]. Moreover, in the NHS, when compared to one serving/d of red meat, one serving/d of poultry was associated with a 19% lower risk of CHD [86]. Additionally, the Costa Rica Heart Study, a population-based case-control study showed lower odds of myocardial infarction when substituting 50 g of chicken without skin and without fat for total red meat (25% reduction), unprocessed red meat (11% reduction), and processed red meat (41% reduction [87].

In contrast, as part of the Lifetime Risk Pooling Project, data were pooled from six prospective US cohort studies comprising the ARIC Study, CARDIA (Coronary Artery Risk Development in Young Adults) Study, CHS (Cardiovascular Health Study), FHS (Framingham Heart Study), FOS (Framingham Offspring Study), and MESA (Multi-Ethnic Study of Atherosclerosis). Findings showed each additional two servings/week of poultry was associated with a 4% increase in incident CVD [88]. Additionally, evidence from 474,985 middle-aged men and women in the UK Biobank study showed that an increase of 30 g/d of poultry consumption was associated with an 8% increased risk for ischemic heart disease over an eight-year follow-up period [89].

Substitution analysis is an important nutrition research approach providing insights into the impacts of different foods on health outcomes. A meta-analysis by Papp et al. [2] showed a 29% decrease for CVD when substituting poultry for processed meat, and a neutral association when substituting (per 100 g/d) poultry for red meat or unprocessed red meat [2]. For CHD, substituting poultry for red meat predicted a 17% decreased risk for CHD and a neutral association when substituting poultry for processed meat or red and processed meat [2]. These observations suggest that the replacement of processed meat or red meat with poultry may reduce the risk for CVD and CHD, respectively. However, these findings should be interpreted with caution as based on the GRADE approach, the certainty of evidence was rated as low or very low. As such, more prospective cohort studies investigating associations of substituting processed or red meat with poultry are warranted.

#### 7.2.2. Stroke

Evidence from observational studies indicates that poultry consumption has a neutral or beneficial association with stroke morbidity and mortality.

The 2021 systematic review and meta-analysis by Papp et al. [2] showed no association between the highest and lowest intakes of poultry and stroke based on nine studies. In addition, both linear and non-linear dose response meta-analysis showed no association between poultry consumption and stroke based on nine studies [2]. These findings should be interpreted with caution, due to the certainty of evidence being deemed weak for stroke using the GRADE approach. Another dose−response meta-analysis of seven prospective cohort studies assessing poultry intake and the risk of stroke showed no association for poultry intake and total stroke risk or risk of subtypes of stroke, namely, ischemic and hemorrhagic stroke [90]. In line with these observations, evidence from The JACC Study [81] and The Hiroshima/Nagasaki Life Span Study [91] showed no associations between intakes of poultry or chicken, respectively, and stroke mortality [81,91]. Evidence from both the UK Biobank study [89] and the EPIC cohort including participants from nine countries in Europe [92] found no associations between poultry intake and ischemic or hemorrhagic stroke [89,92].

Results from some prospective cohort studies indicate an inverse association between poultry consumption and stroke. A meta-analysis of prospective cohort studies showed a 13% reduction in stroke incidence when comparing the highest vs. the lowest categories of intake [93]. Analyses of women in the NHS and men from the Health Professionals Follow-up Study (HPFS) showed a dose−response association between poultry consumption and a 13% reduction in risk of stroke for men and women combined for the highest compared to the lowest quintiles of intake [94]. However, it is important to note that this decreased risk of stroke for men and women combined was largely driven by women, with a more pronounced 18% risk reduction in women alone, whereas in men alone no association was observed [94].

#### 7.2.3. Hypertension

Evidence indicates that poultry consumption is associated with either an increase in blood pressure or neutral association with blood pressure.

Among prospective cohort studies, a meta-analysis of six studies found greater poultry consumption was associated with increased risk for hypertension: 15% greater risk for highest vs. lowest intake groups [95]. Three longitudinal cohort studies in the US—NHS I, NHS II, and HPFS—investigated the relation between long-term intake of animal meats with incident hypertension. Long term, consuming more poultry (≥1 serving/day vs. <1 serving/month of chicken and turkey, with or without skin) was associated with a 22% increased risk of hypertension [96]. The INTERnational study on MAcro/micronutrients and blood Pressure (INTERMAP), which included men and women ages 40–59 years from 17 population samples in the US, United Kingdom, China, and Japan, showed that unprocessed poultry was associated with a higher systolic blood pressure by +0.73 mmHg, but not diastolic blood pressure, in the Western population [97]. The Chicago Western Electric Study in men, showed that the consumption of >20 servings (120 g/serving)/month compared to <4 servings/month, was associated with both higher systolic and diastolic blood pressures [98].

In contrast to these findings, both the CARDIA Study and the Tehran Lipid and Glucose Study showed no associations between poultry intake and 15-year [99] or 3-year [100] incidence of elevated blood pressure. In addition, among older women in the WHI [101] and middle-aged men in Japan [102], no associations between poultry/chicken consumption and blood pressures were observed [101,102].

## 8. Poultry Consumption and T2DM

Type 2 diabetes mellitus is a chronic metabolic condition affecting how the body regulates and processes glucose. Type 2 diabetes mellitus is the third leading cause of diet-related deaths worldwide and the eighth-leading cause of deaths in the US, accounting for 338,700 [70] and 102,188 [103] deaths in 2017 and 2020, respectively. The number of adults with T2DM worldwide has nearly quadrupled in the last four decades, according to the World Health Organization [104]. In the US, the number of adults diagnosed with T2DM has more than doubled in the last 20 years [103]. In the US, it is estimated that more than 32.4 million adults—approximately one in ten—have T2DM. An additional 88 million adults—greater than one in three adults—have prediabetes (when blood glucose concentrations are above the normal range but not above the threshold for a diagnosis of T2DM). Additionally, one in five people do not realize they have T2DM, while more than eight in ten do not realize they have prediabetes, negating treatment and prevention therapies [74,103].

Compared to adults without T2DM, adults with T2DM are 1.7 times more likely to die of cardiovascular-related deaths. In addition, T2DM can cause serious complications such as CVD, and T2DM is the number one cause of kidney failure, lower-limb amputations, and blindness. T2DM also comes with a significant economic burden: direct medical costs for T2DM were estimated to be $237 billion, and an additional $90 billion in indirect costs, in 2020 [103].

### 8.1. RCTs

Evidence from RCTs indicates that when compared to the consumption of solely a carbohydrate source, the combined ingestion of chicken or turkey with a carbohydrate source may result in beneficial effects on indices of glycemic control.

#### 8.1.1. Acute Feeding RCTs

Two randomized cross-over controlled trials investigated the effects of lean chicken breast consumption (22–25 g of protein from chicken) on the insulinemic and glycemic responses to white rice (providing 50 g of carbohydrates) in healthy adults, with inconsistent results [105,106]. Sun et al. [105] reported that compared to white rice alone, the combination of white rice and 22.5 g of protein from chicken breast without skin lowered peak glucose and glucose incremental area under the curve (iAUC) over 120 min by 9% and 26%, respectively. The combination of rice and chicken breast also resulted in greater peak insulin and iAUC over 120 min by 22% and 30%, respectively. Quek et al. [106] reported no differences in glycemic or insulinemic responses (peak or iAUC over 120 min for both) to consuming white rice vs. white rice plus 25 g of protein from chicken breast for glycemic or insulinemic responses [106].

Similarly, another randomized cross-over trial investigated the effects of chicken breast on the glycemic and insulinemic responses to 50 g of carbohydrate from mashed potatoes [107]. Chicken breast (providing 30 g of protein) combined with potatoes, compared to potatoes alone, resulted in lower blood glucose 30 min post-ingestion by 30%, a 42% reduction in glucose iAUC, with no differences in insulinemic responses [107].

In a randomized cross-over trial involving 17 males characterized as older (mean age ± SD of 63 ± 2 years) and overweight with T2DM were assigned to consume 50 g of glucose alone or 50 g of glucose plus 25 g of protein from turkey, among other protein sources (lean beef, gelatin, egg white, cottage cheese, fish, or soy) [108]. The 5 h iAUC for glucose and insulin for the co-ingestion of glucose plus a protein source (except for egg white) were lower and higher, respectively, compared to glucose alone [108]. The findings suggest that the co-ingestion of a protein source, inclusive of turkey, with glucose can decrease the glycemic response and increase the insulinemic response in older males classified as overweight and with T2DM.

Taken together, evidence from acute feeding RCTs indicate that the co-ingestion of lean chicken breast or turkey with a carbohydrate source can improve the glycemic response and either increase or have no effect on the insulinemic response in healthy adults or older males classified as overweight and with T2DM.

#### 8.1.2. Chronic Feeding RCTs

Diabetes can lead to reduced kidney function. While higher protein intake does not apparently impair kidney function in heathy adults [109], reduced protein intake is recommended to help manage neuropathy, also referred to as diabetic kidney disease [110,111,112]. In those with T2DM, the inclusion of chicken-based diets is apparently beneficial for renal function [113,114,115]. A randomized, cross-over, controlled trial involving 28 adults with T2DM assessed the effect of replacing red meat with chicken in participants’ usual diets or a low-protein diet on cardiometabolic disease-related outcomes. The outcomes included glomerular filtration rate (GFR), urinary albumin excretion rate (UAER), blood lipids and lipoproteins, blood pressures, and blood glucose [113]. The participants were assigned to consume a low-protein diet (0.5–0.8 g/kg/d), their usual diet including red meat, or a chicken-based diet (skinless leg quarter) for periods of four weeks each, with a four-week washout between each intervention diet. Total energy intake was not different between the chicken and red meat diets, but there was a 10% lower total energy intake in the low-protein diet compared to chicken and red meat diets. Protein intake was higher in the chicken diet (1.35 g/kg/d) and usual diet with red meat (1.43 g/kg/d), compared to the low-protein diet (0.66 g/kg/d). For the analyses, participants who were normo-albuminuric (24 h UAER < 20 μg/min) and micro-albuminuric (24 h UAER 20–200 μg/min) were analyzed separately [113].

In participants classified as normo-albuminuric, GFR following the chicken and low-protein diets were 11% and 17% lower, respectively, vs. usual diet with red meat. There were no differences in GFR between the low-protein and chicken diets. Consuming the varied diets did not differentially affect UAER, measures of glycemic control, blood lipids and lipoproteins, or blood pressure among diets in normo-albuminuric participants. [113]. In participants classified as micro-albuminuric, GFR was lower by 9% and 13%, respectively, with the low-protein diet compared to the usual diet with red meat and chicken diet. UAER was lower for the chicken diet compared to the low-protein diet or usual diet with red meat by 34% and 46%. Total cholesterol and apolipoprotein B were lower for the chicken diet by 12% and 15%, respectively, and low-protein diet by 13% and 23%, respectively, compared to the usual diet with red meat. No differences in other blood lipids and lipoproteins, measures of glycemic control, or blood pressure were reported in micro-albuminuric participants [113]. These findings may suggest that incorporating lean chicken in place of red meat in a diet may represent an alternative strategy to a low protein diet for adults with T2DM to manage their renal function.

Consistent with these findings, a randomized, cross-over, controlled trial including 17 older adults (mean age ± SD of 59 ± 11 years) with T2DM and macro-albuminuria (24 h UAER ≥ 200 μg/min), found that UAER and non-HDL cholesterol were lower after the chicken diet by 14% and 7%, respectively, and low-protein diet by 27% and 7%, respectively, vs. the red meat diet. [114]. While both short-term studies [113,114] provide evidence that chicken consumption, in place of red meat, may be beneficial for renal function and some blood lipid and lipoprotein measures in individuals with T2DM, there is still the question of longer-term effects.

To address this question, Mello et al. [115] conducted a randomized, open-label, controlled clinical trial with a follow-up period of one year. They assessed the effects of consuming a chicken-based diet plus placebo compared to the angiotensin-converting enzyme inhibitor enalapril (10 mg/d) on renal function and lipid profile in 28 older aged (mean age ± SD of 54.1 ± 10.9 years) participants with T2DM and microalbuminuria. Energy and macronutrient intakes were not different between groups, with total protein intake 1.2–1.3 g/kg/d. Following one year, both diets reduced UAER and mean blood pressure, while the chicken diet reduced total and LDL cholesterol. Participants in the enalapril group had reduced GFR, with a trend for reduced GFR in the chicken group (*p* = 0.069) [115].

Taken together, evidence from these chronic feeding RCTs suggests adults with T2DM who consume chicken as the primary protein source, may experience relatively favorable changes in UAER and lipid profile, with no adverse effects on glycemic control.

### 8.2. Observational Studies

Observational studies on chicken/poultry consumption and diabetes risk, morbidity, and mortality provide inconsistent findings.

Two meta-analyses including 28 prospective cohort studies (accounting for duplicates) showed no associations between poultry intake and risk of T2DM [116,117]. The EPIC-InterAct Study, a case-cohort study including 340,234 adults with a 11.7-year follow-up, observed no association between poultry consumption and risk of T2DM [118]. In contrast, evidence from the UK Biobank study showed 30 g/d greater poultry consumption was associated with a 14% increased risk for diabetes over an eight-year follow-up period [89]. The Singapore Chinese Health Study, a prospective cohort study with 63,257 participants and 11-year follow-up, showed that when comparing the highest to the lowest quintiles of poultry intake, higher intakes were associated with a 15% increased risk of developing T2DM [119].

Results from several observational studies show associations between greater poultry intake and decreased risk for T2DM. The SWHS, a prospective cohort study including 74,493 middle- and older-aged women found that greater unprocessed poultry intake was associated with a decreased risk of T2DM [120]. Interestingly, the association between poultry intake and risk of developing T2DM may be modified by body weight: greater poultry intake was related to a decrease in T2DM risk for those classified as normal weight, but not for those classified with obesity [120]. Prospective evidence from the Alpha-Tocopherol, Beta-Carotene Cancer Prevention Study (ATBC) cohort also showed a reduced risk of T2DM with higher intakes of poultry [121]. Evidence from the EPIC study using a case-cohort design showed a decreased risk of T2DM mortality with higher intakes of poultry in individuals with T2DM [122].

In observational studies, there is a dearth of information assessing processed poultry intake or distinguishing between unprocessed and processed poultry. Our scoping review identified that only four of 366 (1%) observational studies assessed the influence of processed poultry on human health outcomes [4]. Steinbrecher et al. [105] using prospective data from the Hawaii population of the Multiethnic Cohort (MEC) Study, assessed associations between fresh poultry or processed poultry intake and risk of T2DM. The authors reported no association between fresh poultry consumption and T2DM risk. However, when comparing the highest vs. the lowest quintiles of processed poultry intake (≥1.81 vs. 0.04 g/1000 kcal/d) there was a 30% increased risk for T2DM [105]. These observations underscore the need to assess unprocessed and processed poultry separately to enhance understanding of how they influence risk for T2DM.

The substitution of poultry for red meat indicates either an inverse or neutral association with T2DM. A pooled analysis of three prospective cohort studies—the HPFS, the NHS, and the NHS II—showed that the substitution of one serving/d (4–6 oz/d) of poultry for total red meat, unprocessed red meat, or processed red meat was associated with a 15%, 15%, and 22% decreased risk for T2DM, respectively [123]. Using data from the EPIC-InterAct case cohort, a neutral association was observed when substituting 50 g/d of poultry for red or processed meat [124]. Similarly, using data from the Danish Diet, Cancer and Health study, a neutral association was observed for substituting 150 g/week of poultry for total red meat and unprocessed red meat. A 4% decreased risk for T2DM was observed when substituting poultry for processed red meat [125].

## 9. Conclusions

Total chicken and poultry intakes in the US have increased over time, tripling from 1960 to 2022. The nutritional composition of chicken and other poultry products varies depending on the cut, leanness, and processing level of the meat. Chicken and other poultry products are sources of high-quality dietary protein and other essential nutrients required for human health and physiological functioning, in relatively high amounts. Chicken and other poultry products may also have other nutritive and non-nutritive components such as saturated fat, sodium, and nitrites, depending on the cut of poultry meat and/or level of processing, that should be consumed with caution. Cooking methods of chicken and other poultry products also introduce variability in the concentration of non-nutritive compounds. High-heat cooking methods can result in the formation of compounds such as advanced glycation end-products, heterocyclic aromatic amines, and polycyclic aromatic hydrocarbons that are associated with negative cardiometabolic health-related outcomes. Therefore, the nutritive and non-nutritive components of chicken and other poultry products can contribute to human health in positive and negative ways.

An important consideration regarding experimental and observational studies is that inconsistencies in the findings may, in part, be explained by discrepant definitions of poultry, a lack of distinction and specific categorizations of chicken/poultry products (e.g., unprocessed vs. processed), amounts and types consumed, and cooking methods, which influence the energy and nutrient profiles and health-related outcomes. It is also important to emphasize that the conclusions provided below apply to the specific types of poultry products included in the articles synthesized in this review and that other dietary and lifestyle factors also influence health outcomes.

Limited evidence from RCTs suggest consuming lean unprocessed animal-based protein-rich foods, inclusive of chicken, as the primary source of dietary protein favorably affects body weight or body composition concurrent with purposeful weight loss, but not weight maintenance. Observational studies provide inconsistent findings that greater chicken/poultry consumption is either unrelated or positively related to higher BMI.

Pertaining to CVD, the consumption of varying lean meats (poultry, red meat, fish) as a primary protein food source does not influence the health-promoting effects of consuming a healthy dietary pattern on multiple CVD risk factors. These findings are supported by a meta-analysis of RCTs showing no differential effects between red meat and poultry consumption on CVD risk factors [126]. In “generally healthy” adults, when the primary sources of protein foods consumed as part of a typical American diet are nonmeat, reductions in blood lipids and lipoproteins can occur compared to lean poultry or red meat. This should be interpreted with caution, due to the limited number of RCTs specifically pertaining to chicken/poultry consumption and CVD risk factors.

Evidence from observational studies indicates greater poultry consumption has either neutral or beneficial associations with CVD, CHD, ischemic heart disease, and stroke, but neutral or adverse associations with blood pressure and hypertension.

Pertaining to T2DM, results from RCTs and observational studies assessing the effects of or associations between chicken/poultry intake and T2DM seem inconsistent. Evidence from RCTs indicate the consumption of lean chicken/poultry is either beneficial or has neutral effects on T2DM risk factors in healthy individuals, those at an increased risk for T2DM, and those with T2DM. In acute feeding trials, co-ingestion of chicken/poultry with a carbohydrate source favorably affects glycemic responses, with favorable or neutral effects on insulinemic responses. Regarding renal function, lower protein diets are being used to help manage diabetic kidney disease. Limited provocative evidence suggests higher-protein intakes achieved with lean chicken as the primary dietary protein source, may have relatively beneficial or neutral effects on renal function in those with T2DM. Healthy adults may consume higher protein diets without detrimental effects on renal function. Inconsistent evidence from observational studies may be used to support beneficial, neutral, or detrimental associations between chicken/poultry consumption and T2DM-related outcomes.

### Future Research Recommendations

Based on evidence identified from our comprehensive systematically searched scoping review [4] and synthesized in this narrative review on poultry consumption and body weight and body composition, CVD, or T2DM health-related outcomes, the following suggestions may be considered for future research:

(1) There is a need for future experimental and observational research to include definitions and detailed descriptions of the different types and amounts of poultry products consumed.
  Rationale: The research that currently exists on poultry is complicated by the lack of a clear definition of poultry and limited descriptions of the types and forms of poultry used among studies. Importantly, “muscle food categories and descriptions are substantively different within and between experimental and observational studies and do not match regulatory definitions” [20]. New research with greater consideration and more detailed descriptions of multiple factors, including the amounts, types, and forms of chicken/poultry consumed; the health, medical, and dietary characteristics of the research cohorts; and other confounding factors will help improve our understanding of the influence of chicken/poultry on body weight and body composition, CVD, and T2DM.

(2) Chronic feeding RCTs are warranted assessing the effects of consuming unprocessed and processed poultry products in the context of healthy and unhealthy dietary patterns on body weight and body composition, CVD, and T2DM health-related outcomes.
  Rationale: Of 59 RCTs identified in our scoping review [4], only seven included assessments of body weight and body composition, 17 included CVD risk factors, and 11 included T2DM risk factors. Accounting for overlap among studies, there were 26 unique RCTs. Zero RCTs specifically assessed processed chicken/poultry, 23 RCTs assessed unprocessed chicken/poultry, and 3 RCTs were indeterminate. Most chronic feeding RCTs did not document (e.g., using the Healthy Eating Index) the healthfulness of the dietary pattern.

(3) Observational studies are warranted examining associations between processed poultry consumption or poultry cooking methods and body weight and body composition, CVD, or T2DM health-related outcomes in humans across the life course.
  Rationale: Our scoping review identified only four of 366 (1%) observational studies assessed the influence of processed poultry on human health outcomes [4]. Of these four observational studies identified and included in this review, three were on body weight or BMI and one was on T2DM risk. Zero observational studies were identified that included investigating associations between processed poultry consumption and CVD. In addition, only 14% of 366 observational studies reported the cooking method used [4].

## Figures and Tables

**Table 1:** Nutrient contents of selected poultry products [Source: United States Department of Agriculture FoodData Central [7]].

	Values per 100 g Cooked Product														
	Unprocessed Poultry Products											Processed Poultry Products			
Nutrient	Chicken Breast, Skinless, Boneless, Roasted	Dark Chicken Meat, Skinless, Boneless, Roasted	Chicken Drumstick, Skinless, Boneless, Roasted	Chicken Drumstick, Skin-on	Chicken Thigh, Skinless	Chicken Thigh, Skin-on, Roasted	Chicken Wing, Skinless	Chicken Wing, Skin-on, Roasted	Ground Turkey, 93% Lean, 7% Fat, Pan-Broiled	Turkey Patty, 93%, 7% Fat, Broiled	Turkey patty, 85%, 15% fat, broiled	Chicken Nuggets, White Meat, Breaded, Precooked, Frozen	Chicken Tenders, Breaded, Precooked, Frozen	Chicken, Luncheon Meat	Turkey, Luncheon Meat
Calories (kcal)	165	205	155	191	179	232	203	254	213	207	249	261	240	98	106
Protein (g)	31.0	27.4	24.2	23.4	24.8	23.3	30.5	23.8	27.1	25.9	25.9	14.4	14.6	17.4	14.8
Total fat (g)	3.6	9.7	5.7	10.2	8.2	14.7	8.1	16.9	11.6	11.4	16.2	15.4	13.6	1.9	3.8
Saturated fat (g)	1.0	2.7	2.1	2.7	2.3	4.1	2.3	5.0	3.0	3.0	4.1	3.4	2.4	0.6	0.9
Monounsaturated fat (g)	1.2	3.6	3.1	4.2	3.4	6.3	2.6	7.7	3.8	3.9	5.5	4.7	4.1	0.7	1.1
Polyunsaturated fat (g)	0.8	2.3	1.6	2.1	1.7	3.0	1.8	3.6	3.5	3.5	4.2	6.2	6.2	0.5	1.0
Carbohydrates (g)	0.0	0.0	0.0	0.0	0.0	0.0	0.0	0.0	0.0	0.0	0.0	16.2	14.9	0.0	0.0
Cholesterol (mg)	85	93	128	130	133	133	85	141	104	106	105	34	36	51	49
Sodium (mg)	74	93	128	123	106	102	92	98	90	91	81	538	527	1032	898
Vitamin D (µg)	0.1	0.1	0.1	0.1	0.2	0.2	0.1	0.2	0.2	0.2	0.2	0.1	0.1	0.1	0.2
Calcium (mg)	15	15	11	11	9	9	16	18	31	29	48	38	39	11	14
Iron (mg)	1.0	1.3	1.1	1.1	1.1	1.1	1.2	0.8	1.6	1.7	2.0	1.4	0.8	0.4	0.4
Potassium (mg)	256	240	256	247	269	253	210	212	304	247	242	281	281	360	371
Vitamin B2 [riboflavin] (mg)	0.1	0.2	0.2	0.2	0.2	0.2	0.1	0.2	0.3	0.2	0	0.1	0.1	0.1	0.1
Vitamin B3 [niacin] (mg)	13.7	6.5	5.6	5.4	6.2	5.8	7.3	6.3	8.1	6.6	7	6.7	5.9	9.1	7.2
Vitamin B5 [pantothenic acid] (mg)	1.0	1.2	1.1	1.1	1.3	1.2	1.0	0.9	1.4	1.3	1	0.9	0.9	1.0	0.6
Vitamin B6 (mg)	0.6	0.4	0.4	0.4	0.5	0.4	0.6	0.6	0.5	0.5	0.4	0.3	0.3	0.4	0.4
Vitamin B12 (µg)	0.3	0.3	0.4	0.4	0.4	0.4	0.3	0.4	1.9	1.8	1.4	0.2	0.2	0.1	0.4
Phosphorus (mg)	228	179	205	200	230	216	166	147	259	210	235	213	200	257	249
Magnesium (mg)	29	23	24	22	24	22	21	19	29	25	25	35	29	26	19
Zinc (mg)	1.0	2.8	2.6	2.4	1.9	1.7	2.1	1.6	3.8	3.7	3.3	0.8	0.6	0.5	0.9
Selenium (µg)	27.6	18.0	25.2	28.1	27.1	25.3	24.7	25.5	28.4	27.5	35.1	17.0	19.3	13.2	13.0
Choline (mg)	85.3	74.0	63.4	67.8	71.8	67.6	79.6	111.3	78.7	77.7	79.1	45.5	46.1	44.2	30.1

## Data Availability

No new data were created or analyzed in this study. Data sharing is not applicable to this article.
